# Comparative Efficacy of Palonosetron–Dexamethasone Versus Granisetron–Dexamethasone for Prevention of Postoperative Nausea and Vomiting: A Review With Focus on Otologic Surgeries

**DOI:** 10.7759/cureus.104098

**Published:** 2026-02-23

**Authors:** Khushboo Bairwa, Richa Kewalramani, Anuradha Vaswani, Mukesh Somvanshi, Sandeep Bairwa

**Affiliations:** 1 Department of Anesthesiology, Kshetrapal Hospital Multispeciality and Research Hospital, Ajmer, IND; 2 Department of Anesthesiology and Critical Care, All India Institute of Medical Sciences, Jodhpur, Jodhpur, IND; 3 Department of Anesthesiology, Government Medical College, Kota, Kota, IND; 4 Department of Medical Oncology, Kshetrapal Hospital Multispeciality and Research Hospital, Ajmer, IND

**Keywords:** antiemetic prophylaxis, corticosteroids, otologic surgery, postoperative nausea and vomiting, prophylaxis

## Abstract

Postoperative nausea and vomiting (PONV) remain one of the most distressing and consequential complications in perioperative care, with a particularly high incidence in otologic surgeries, where vomiting can jeopardize graft stability and hearing outcomes. This review provides a focused, evidence-based evaluation of the comparative efficacy of palonosetron-dexamethasone and granisetron-dexamethasone combinations in preventing PONV, integrating pharmacologic insights with procedure-specific clinical implications. Drawing upon studies published between 2015 and 2025, it highlights the superior and sustained efficacy of palonosetron, a second-generation serotonin (5-hydroxytryptamine type 3; 5-HT₃) receptor antagonist, due to its longer half-life, allosteric receptor binding, and enhanced pharmacodynamic stability. The synergistic benefit of combining a 5-HT₃ receptor antagonist with dexamethasone, a corticosteroid with anti-inflammatory and central inhibitory properties, is emphasized, demonstrating improved control of both early and delayed PONV with minimal adverse effects. Moreover, the review contextualizes these findings within the unique challenges of otologic procedures and discusses their safety, cost-effectiveness, and clinical applicability. By bridging pharmacologic evidence with surgical realities, this review underscores the need for procedure-specific, long-acting antiemetic strategies and advocates for multicentric, cost-conscious research to refine prophylactic protocols. Ultimately, it offers a novel synthesis that advances PONV management in otologic surgery through the integration of clinical efficacy, patient safety, and economic prudence. The evidence supports palonosetron-dexamethasone as the more effective and sustained prophylactic regimen compared with granisetron-dexamethasone, particularly for delayed PONV (6-48 h) in otologic surgeries. Palonosetron-based dual therapy should be preferred in high-risk middle ear procedures where postoperative vomiting may compromise graft integrity, while granisetron-dexamethasone remains a reasonable lower-cost option for shorter or lower-risk cases.

## Introduction and background

Postoperative nausea and vomiting (PONV) remain one of the most common and unpleasant post-anesthesia issues [1]. The middle ear surgeries have a high rate of PONV, with up to 80 percent of patients experiencing the condition despite improvements in perioperative medicine [2]. What is especially alarming about PONV is the fact that, in addition to the discomfort, vomiting or even retching may lead to the displacement of sensitive grafts and surgical repairs and, therefore, a loss of hearing [3]. So, the prevention of PONV during middle ear surgeries is not only a parameter of patient satisfaction but also an essential factor of surgical success [4].

The pathophysiology of PONV is complicated and includes both the activation of the chemoreceptor trigger zone (CTZ) and the vomiting center in the medulla and the input of the gastrointestinal tract, the vestibular system, and the higher cortical centers. These neurotransmitters include serotonin (5-hydroxytryptamine; 5-HT), dopamine, histamine, acetylcholine, and substance P, which attach to the corresponding receptors (5-HT_3_, D2, H1, M1, and NK1) to regulate these pathways, which eventually lead to the vomiting reflex [6]. The PONV predisposition is a varied phenomenon, and it relies on numerous risk factors, such as female sex, non-smoking status, a prior history of PONV or motion sickness, and the need for postoperative opioid analgesia. The risk of PONV increases progressively with the number of risk factors present. Apfel et al. [7] proposed a simplified risk score predicting an approximate PONV risk of 10% with no risk factors, 20% with one, 40% with two, 60% with three, and 80% when all four factors are present. In addition, younger age (commonly defined as <50 years) has been identified as an independent risk factor, particularly for postdischarge nausea and vomiting [7]. Such risk is enhanced by middle ear manipulation and vestibular stimulation and increased by the use of volatile anesthetics, nitrous oxide [8], and perioperative opioids, which increase the emetogenic burden [9]. This compound interaction is the reason why none of the antiemetics can provide complete protection [10].

**Figure 1:**
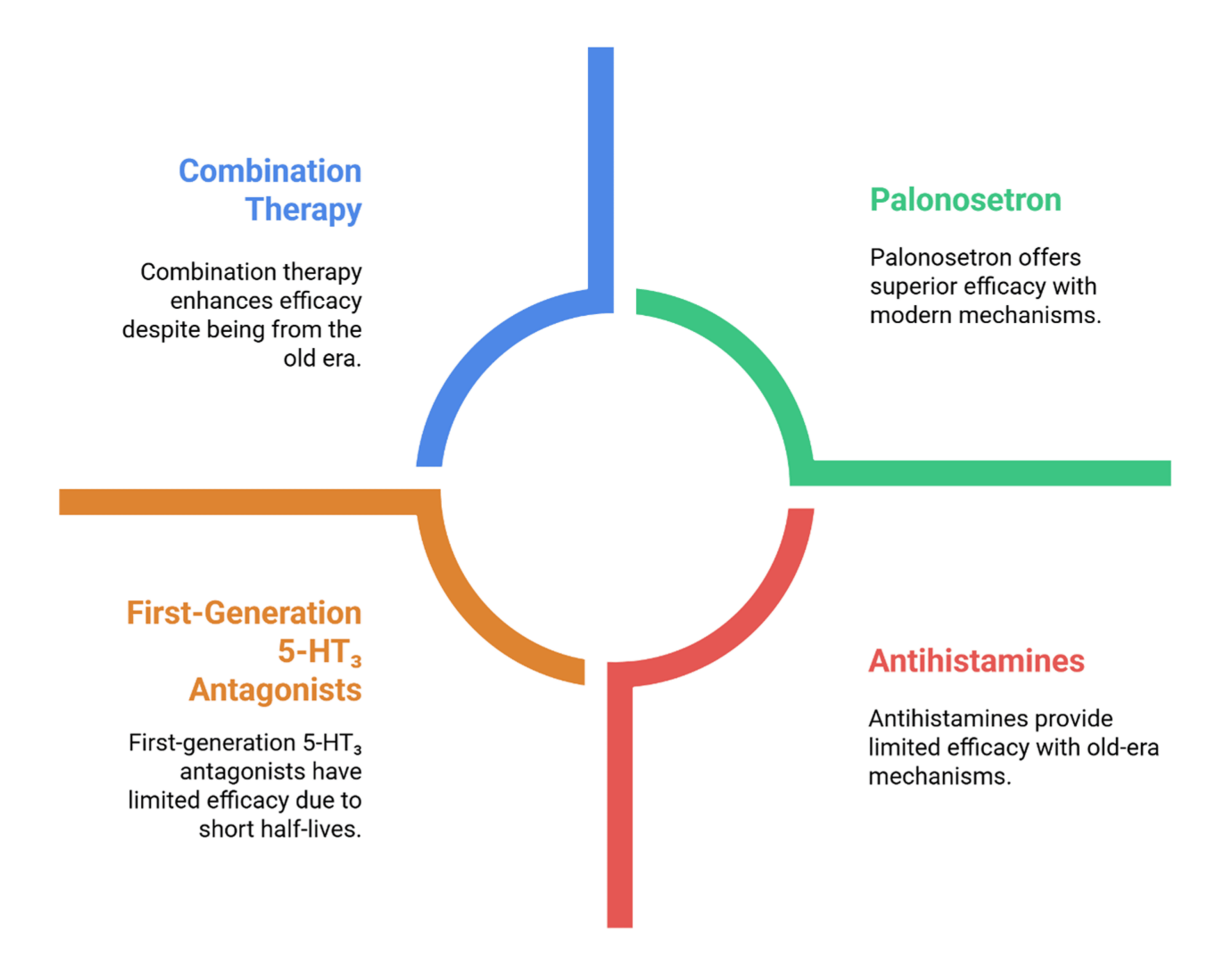
Comparative efficacy of antiemetic strategies Created by authors

The prophylaxis against PONV in the past was based on antihistamines, anticholinergics, and dopamine antagonists, which had limited efficacy and were usually accompanied by side effects like sedation, extrapyramidal side effects, and dry mouth [10]. The development of 5-HT_3_ receptor antagonists was an important step with a direct focus on serotonergic pathways in the gastrointestinal tract and the central nervous system [11]. These agents prevent the binding of serotonin to vagal afferents and CTZ and thus disrupt a major emetic pathway [12]. The first-generation drugs ondansetron, granisetron, and dolasetron proved to be effective and formed the basis of current PONV prevention regimens [13]. The relative effectiveness of antiemetic regimens between traditional agents, combination therapy, and palonosetron is shown in Figure 1.

Nevertheless, the first-generation agents have one significant drawback: they do not last as long (have shorter plasma half-lives) in treating delayed PONV, which normally follows surgery 6-48 hours later [14]. As an example, granisetron has a half-life of about nine hours, and in most cases, it does not cover the entire period of PONV during surgery [15]. This gap was filled by the development of palonosetron, a second-generation 5-HT_3_ antagonist, which has distinct pharmacologic characteristics, including allosteric receptor binding, receptor internalization, and a long half-life of almost 40 hours [16]. Comparative clinical trials in abdominal, gynecologic, and orthopedic surgery have repeatedly shown that palonosetron is more effective than placebo in the prevention of early and delayed PONV [17].

The steroid glucocorticoid dexamethasone has been added to the repertoire of multimodal PONV prophylaxis alongside serotonin antagonism [18]. Despite the fact that the exact mechanism of action has not been fully determined, dexamethasone is believed to work by inhibiting the production of prostaglandins, suppressing inflammation of the central nervous system, and altering the neurotransmission within the nucleus tractus solitarius [19]. Dexamethasone can be used in combination with a 5-HT_3_ antagonist, which has synergistic antiemetic effects and acts on more than one emetogenic pathway at once [20]. Consistent meta-analyses and clinical trials demonstrate that such combination therapy, in particular, the 5-HT_3_ antagonists that are combined with corticosteroids, has better protection than single-drug regimens. This has led to a majority of the clinical guidelines suggesting the use of dual prophylaxis in high-risk patients, even when they are undergoing middle ear surgery.

It is commonly used in two combinations: granisetron and granisetron-dexamethasone and palonosetron. They are both clinically effective, but due to pharmacokinetic benefits, palonosetron offers long-term protection, making it useful in delayed postoperative periods. This difference is especially relevant to the field of otologic surgery, where structural surgical complications may occur because of PONV. Considering such clinical implications, careful, comparative analysis of granisetron-based and palonosetron-based regimens is a necessary solution to maximizing antiemetic regimens and enhancing the success rate of middle ear surgery.

Objectives of the review

This review provides a critical and comprehensive evaluation of the literature on serotonin receptor antagonists, focusing on palonosetron and granisetron in combination with corticosteroids for the prevention of PONV. It compares pharmacologic characteristics, clinical efficacy, and safety using evidence from randomized controlled trials, systematic reviews, and meta-analyses. Special emphasis is placed on their use in middle ear and other otologic surgeries. The review aims to guide evidence-based, procedure-specific antiemetic selection while identifying gaps for future research.

Methodology

This review employed a narrative and comparative literature review framework to aggregate the latest evidence regarding the use of serotonin receptor antagonists plus corticosteroids to prevent PONV, especially after otologic and middle ear surgeries. Structured searches in PubMed, Scopus, Google Scholar, and the Cureus databases helped to retrieve the relevant articles. The investigated keywords and their combinations were as follows: "postoperative nausea and vomiting," "palonosetron," "granisetron," "dexamethasone," "5-HT antagonists," "middle ear surgery," and "otologic surgery." Search results were refined, and comparative and overlapping pieces of evidence were captured through the use of Boolean operators (AND/OR).

The studies involved in this review were published within the period of 2015 and 2025, were in English, and were randomized controlled trials, systematic reviews, meta-analyses, and clinically relevant narrative reviews. The studies included the use of 5-hydroxytryptamine type 3 (5-HT_3_) receptor antagonists, used alone or in combination with corticosteroids or other adjuvant agents, including dexamethasone, midazolam, dexmedetomidine, haloperidol, or aprepitant, in articles that addressed pharmacology, mechanisms, or clinical management of PONV were also taken into consideration. Articles were eliminated when they were not within the set time and language parameters, when they were not peer-reviewed or clinically relevant, or when they were not in relation to surgical or perioperative settings in relation to PONV management.

The review team took out the information related to the study design, participant characteristics, interventions, dosage regimens, and outcome measures with specific emphasis on PONV incidence and severity, the use of rescue antiemetics, and the incidence of adverse events. Preference was given to trials comparing combinations of palonosetron-dexamethasone and granisetron-dexamethasone and otologic or middle ear surgery. All the obtained information was qualitatively synthesized to identify the pharmacological peculiarities, clinical efficacy patterns, and the best perioperative antiemetic prophylaxis according to the canons of evidence-based medicine.

## Review

Pathophysiology and risk factors of PONV

PONV is a polyphasic complication that occurs due to the interplay of neural and chemical systems that mediate the emetic reflex [21]. The vomiting center in the medulla oblongata coordinates vomiting, input of which is given by the CTZ, the vestibular system, the higher cortical centers, and the gastrointestinal tract through the vagus nerve [3]. The CTZ is found beyond the blood-brain barrier; it identifies those emetogenic substances that are in circulation, like anesthetics, opioids, and metabolic byproducts [22]. After stimulation, it sends a signal to the vomiting center, which triggers the autonomic and motor responses leading to emesis [4].

It is a very complicated process and is accompanied by various neurotransmitters, such as serotonin (5-hydroxytryptamine; 5-HT), dopamine, histamine, acetylcholine, and substance P [23]. They both stimulate emetic responses via different receptor systems: 5-HT_3_, D2, H1, M1, and NK1 [10]. Among them, serotonin plays a crucial role; it is discharged out of enterochromaffin cells in the gut due to surgical stress, and the signal is delivered to the 5-HT_3_ receptors in the vagal afferents, transmitting the signals to the CTZ and the vomiting center of the brainstem [15]. The association of the vestibular system with nausea occurs via H₁ and M₁ receptors, and this explains why middle ear surgery, which directly activates the vestibular apparatus, has one of the highest PONV risks [8]. This means that the multimodal prophylaxis that involves several receptor systems tends to be inefficient within single-agent antiemetic therapy, resulting in the use of multimodal prophylaxis [24].

**Figure 2:**
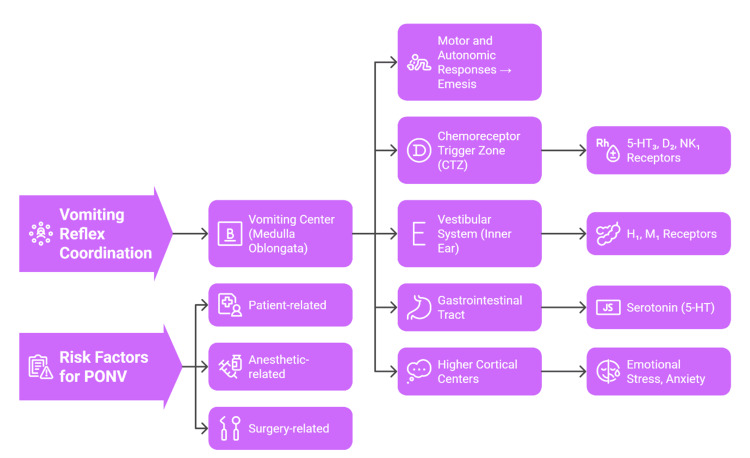
Neural and receptor pathways underlying postoperative nausea and vomiting Created by authors

Patient, anesthetic, and surgical factors play a role in the vulnerability to PONV [25]. Significant risk factors related to patients are female sex, non-smoking status, a prior history of PONV or motion sickness, and the anticipated need for postoperative opioids. The probability of developing PONV increases with the number of risk factors present. Apfel et al. developed a simplified risk score estimating a PONV incidence of approximately 10% in patients with no risk factors, 20% with one, 40% with two, 60% with three, and 80% when all four factors are present. Younger age, commonly defined as <50 years, has also been identified as an independent risk factor, particularly for postdischarge nausea and vomiting [7]. Volatile anesthetics, nitrous oxide, and perioperative opioids increase emetogenic potential through enhancing serotonin release or sensitizing central pathways [26]. Some surgeries, especially the otologic surgeries and ophthalmic surgeries, also increase the risk because of the stimulation of the vault and the increased time of anesthesia. The identification and classification of these risk factors enable clinicians to use individualized evidence-based prophylaxis regimens, a key element in the current anesthetic practice [20]. Figure 2 shows the neural mechanisms and interplay of receptors during PONV.

Historical approaches to PONV management

The approaches to the management of PONV have developed from empirical therapies to pharmacologic approaches that focus on the receptor [27]. The initial antiemetic treatment was mainly based on antihistamines, anticholinergics, and dopamine antagonists, which had modest effects and were frequently constrained by adverse effects [10]. An example is promethazine, which was found to be effective in reducing the nausea of vestibular origin but caused significant sedation and drowsiness [28]. Likewise, scopolamine, an anticholinergic, which alleviates motion-induced nausea through muscarinic receptor blocking, also commonly causes xerostomia, blurred vision, and confusion [29]. Dopamine receptor (D2) dopamine antagonists like metoclopramide and droperidol were more effective in the treatment of vomiting mediated by the dopamine D2 receptors in the CTZ but were not successful because of the risk of extrapyramidal symptoms and cardiovascular instability [3]. Even though these agents had partial symptomatic effects, their low activity and the presence of adverse effect profiles rendered them incompetent to be used in routine prophylaxis [22].

The discovery of serotonin (5-hydroxytryptamine; 5-HT) as a central neurotransmitter in the emetic process resulted in the emergence of 5-HT₃ receptor antagonists [30]. The late 1980s introduction of ondansetron was a milestone, as it offered the most effective blockade of serotonin-mediated nausea and vomiting at a minimum level of sedation [9]. This was succeeded by granisetron and dolasetron, which had similarly lasting efficacy with a little longer duration of action [31]. These agents disrupt the last emetic pathway by inhibiting the serotonin receptors on vagal afferents and in the CTZ, thus acting to prevent the central emetic loops [17]. Their action turned PONV control from a reactive to a proactive pharmacologic method, reducing the usage of rescue antiemetics significantly [20].

The first-generation 5-HT_3_ antagonists were, however, limited by the short half-lives of elimination, hence limiting their effects during the prolonged postoperative period (6-48 hours) [25]. To address this, second-generation antagonists like palonosetron were created, which have longer receptor binding and higher binding affinity [32]. The allosteric receptor modulation and internalization of palonosetron provide long-term effects that are much longer than the plasma half-life, which makes it very effective in the prevention of PONV in early and delayed periods [16]. This transformation of nonselective, short-acting agents to long-acting and receptor-selective agents is one of the significant milestones in the history of perioperative pharmacology, which can improve patient outcomes with minimal side effects [12].

Pharmacodynamics of 5-HT₃ receptor antagonists

5-HT₃ receptor antagonists exert their therapeutic effect by blocking serotonin-mediated signalling at both peripheral and central levels [33]. During surgical stimulation or drug-induced serotonin release, these agents inhibit 5-HT₃ receptors on vagal afferents in the gut and in the CTZ, thereby preventing emetogenic transmission [8]. They block peripheral vagal input and central neurotransmission, which is the foundation of the contemporary prophylaxis against PONV [20]. For optimal prophylactic efficacy, 5-HT₃ receptor antagonists are recommended to be administered approximately 30 minutes before the conclusion of surgery.

The two drugs that are best researched in this category are granisetron and palonosetron [34]. The initial generation antagonist, granisetron, competitively binds to 5-HT_3_ and possesses an average plasma half-life of approximately nine hours, which offers good but temporary relief [15]. Second-generation drugs, such as palonosetron, in contrast, allosterically bind with a greater affinity and lead to receptor internalisation, which leads to longer receptor inhibition [35]. Having a half-life of about 40 hours, the protection is provided during the early and late PONV periods, which have been confirmed during abdominal surgery, gynecologic surgery, and otologic surgery [16]. Relative trials always prefer palonosetron to granisetron in the reduction of PONV occurrence and decrease of rescue antiemetic consumption [31]. This effect is especially useful in sustained use in middle ear surgery, where delayed vomiting can jeopardize the stability of grafts [36]. The side effects of both agents are mild, such as headache or dizziness, and palonosetron has a reduced possibility of causing QT prolongation [26].

Antiemetic effects are further increased by the synergistic actions of 5-HT_3_ antagonists and corticosteroids, with dexamethasone being the most effective [37]. Dexamethasone suppresses the production of prostaglandins, decreases serotonin release, and alters neurotransmission in the brainstem [24]. When used in combination, these medications offer a wide-spectrum effect by killing both inflammatory and serotonergic pathways [17]. A large number of trials and meta-analyses confirm that the two-drug treatment is much more effective in preventing early and delayed PONV than monotherapy, especially in high-risk surgery like tympanoplasty and mastoidectomy [9]. In spite of the fact that palonosetron-dexamethasone provides a longer coverage duration compared to granisetron-dexamethasone, the selection of regimen may be largely determined by other factors such as cost and availability of the drug [19]. Granisetron should still be used in shorter or resource-limited procedures, but palonosetron is more advantageous for longer coverage in high-risk procedures [14]. Both regimes are believed to be safe, evidence-based, and capable of adjustability to patient risk profiles in addition to institutional procedures [12].

Role of dexamethasone as an adjuvant

Dexamethasone has long been a key to multimodal antiemetic prophylaxis due to its broad spectrum of therapy, long-lasting effect, and high safety profile [38]. It was initially known to have a potent anti-inflammatory effect, and the first effect was observed clinically and later confirmed by research [15]. The action of the drug is peripheral and central: inhibition of the release of inflammatory mediators and synthesis of prostaglandins, which reduces the release of serotonin by the enterochromaffin cells of the gastrointestinal tract [10]. Its core region also acts on corticosteroid receptors and suppresses brain neuron transmission in the nucleus tractus solitarius, which reduces brainstem vomiting center activation [39].

One 8 mg dose administered intravenously should offer up to 36 hours of protection, which coincides with the highest level of postoperative risk. Dexamethasone is effective as an antiemetic at an intravenous dose of 4 mg; however, due to its delayed onset of action, it should be administered as early as possible after induction of anesthesia for optimal prophylactic benefit. Prophylactic dexamethasone has also been shown to reduce postoperative pain and improve the quality of recovery, typically at higher doses (around 8 mg) than those required solely for antiemetic efficacy [40]. Dexamethasone is the best due to minimal sedation and cardiovascular disruption; thus, it fits well in ambulatory and otologic surgical procedures where quick recovery and maintenance of a normal hemodynamic level are critical [7]. In addition, it possesses additional positive effects, including less postoperative fatigue, better appetite, and mild painkilling, which add to faster healing [21]. In middle ear surgery, vomiting and inflammation are controlled effectively to promote graft integration and healing [41]. Most perioperative guidelines have suggested dexamethasone as a primary prophylaxis drug in patients at a high risk of PONV because of its good efficacy and safety profile [42]. Table 1 demonstrates that dexamethasone is an effective and safe prophylaxis with lasting action against PONV due to the combination of peripheral and central actions.

**Table 1:** Pharmacologic profile, mechanisms, and clinical applications of dexamethasone in PONV PONV: Postoperative nausea and vomiting, IV: Intravenous, mg: Milligram, ENT: Ear, nose, and throat

Parameter	Mechanism / Description	Clinical Implication	Advantages	References
Drug Class	Synthetic glucocorticoid corticosteroid	Provides potent anti-inflammatory and antiemetic effects	Wide therapeutic range; safe perioperatively	[38]
Peripheral Mechanism	Inhibits prostaglandin synthesis and inflammatory mediator release; decreases serotonin secretion from enterochromaffin cells	Reduces stimulation of vagal afferents and gastrointestinal-mediated nausea	Effective suppression of early PONV	[10,15]
Central Mechanism	Acts on corticosteroid receptors; inhibits neuronal transmission in the nucleus tractus solitarius	Decreases the activation of the brainstem vomiting centre	Prolonged suppression of the emetic reflex	[39]
Dosage and Duration	A single IV dose of 8 mg provides up to 36 hours of action	Matches the peak postoperative risk period	Long duration from a single administration	[40]
Clinical Applications	Used in otologic, laparoscopic, and ambulatory surgeries	Effective for high PONV-risk procedures	Facilitates rapid recovery	[7,41]
Additional Benefits	Improves appetite, reduces fatigue, provides mild analgesia	Enhances postoperative comfort and healing	Promotes graft integration in ear surgery	[21,41]
Safety and Tolerability	Minimal sedation and cardiovascular effects	Safe for outpatient and ENT patients	Excellent safety profile	[7]
Guideline Position	Recommended first-line prophylactic antiemetic for high-risk PONV patients	Integral to multimodal antiemetic regimens	Supported by current clinical guidelines	[42]

Combination therapy: mechanistic synergy

PONV is a polyfactorial issue, and there is no single drug that can be effective in preventing all the emetogenic pathways [43]. As a result, combination therapy, especially the 5-HT_3_ receptor antagonist using dexamethasone, is the most well-accepted treatment method [10]. They are complementary, with serotonin antagonists inhibiting vagal and central emetic circuits activated by adrenaline and dexamethasone, counteracting inflammatory and prostaglandin-mediated stimuli [22]. When they are combined, they offer more extensive and lasting protection than each individually [44].

Numerous randomized controlled trials and meta-analyses validate the greater effectiveness of dual prophylaxis in the reduction of PONV incidence and severity and in the requirement of rescue antiemetic in the early (0-6 h) and delayed (6-48 h) postoperative periods [25]. The best timing is maximization of the outcome that dexamethasone should be administered at the time of induction, and the 5-HT_3_ antagonist should be used close to the end of the operation to coincide with the two effects [41]. This regime particularly helps in otologic surgeries, where PONV may pose a risk to graft integrity. Tympanoplasty and mastoidectomy studies have indicated more than a 50 percent decrease in PONV with the combination of both agents [45]. Patients heal more quickly, need less follow-up care, and have fewer side effects, which are usually temporary headaches or dizziness [46]. Because of its steady effectiveness and tolerability, this two-drug regimen has become the accepted standard of treatment by major anesthesia societies as a PONV prophylaxis [17].

Comparative clinical trials: granisetron vs. palonosetron

Among 5-HT₃ receptor antagonists, the most significant comparisons have been made between granisetron and palonosetron, representing first- and second-generation classes, respectively [47]. Their main dissimilarities consist of the dynamic of receptors and the effectiveness period [12]. Granisetron works by competitive reversible blockade with a half-life of about nine hours, giving effective early postoperative cover but without expanded delayed-phase control [27]. Conversely, palonosetron shows allosteric receptor binding and receptor internalization leading to long receptor inhibition and a half-life of approximately 40 hours [33]. This special pharmacologic action allows one-dose oral intake to persist in the critical postoperative phase [14].

The comparative clinical trials indicate that palonosetron has better efficacy and a longer duration of action in the prevention of PONV [48]. Randomized controlled trials of middle ear surgeries show that palonosetron-dexamethasone has a significant difference in PONV and complete-response rates than granisetron-dexamethasone (97 vs. 73 percent at 24-48 hours) [31]. Likewise, abdominal and gynecologic surgeries have also recorded similar results, and palonosetron has been shown to be stable and widely applicable [16]. Its longest receptor occupancy and high binding affinity make it a better control of delayed emesis without having repeat dosing in a mechanistic way [35].

The two medications have a good safety profile with mild and temporary side effects like headache or dizziness, and serious incidents, such as QT prolongation, are infrequent, especially with palonosetron [45]. The long-lasting efficacy of the latter also decreases the need to use rescue antiemetics and postoperative monitoring, enhancing the comfort of the patients and clinical outcomes [40]. Palonosetron is expensive to acquire but costs less due to less rescue medication administration and shorter recovery periods in high-risk or long-term surgeries [19].

Risk-stratified drug selection is the most logical clinical solution [9]. Granisetron-dexamethasone is still a relatively cheap and accessible choice to use in short or low-risk procedures, with palonosetron-dexamethasone suggested to be used in higher-risk or longer cases, especially in middle ear surgery, where the stability of the grafts may be threatened even by mild retching [5]. In these processes, the sustained effect of palonosetron provides coverage during the recovery period, reduces the number of physiological complications, and improves patient comfort [20]. Comprehensively, it has been shown that both regimens are safe and effective, whereas palonosetron-dexamethasone is better than others in providing long-term prophylaxis, which is why it is the best evidence-based approach to postoperative management in high-risk surgical patients [46]. Table 2 indicates the pharmacologic and clinical comparison between granisetron and palonosetron in the prevention of PONV.

**Table 2:** Comparison of granisetron and palonosetron in PONV prevention 5-HT₃: 5-Hydroxytryptamine type 3, PONV: Postoperative nausea and vomiting, h: Hours, Ki: Inhibition constant, QT: QT interval on electrocardiogram

Parameter	Granisetron (First-Generation 5-HT₃ Antagonist)	Palonosetron (Second-Generation 5-HT₃ Antagonist)	Clinical Implication	References
Mechanism of Action	Competitive, reversible 5-HT₃ receptor blockade	Allosteric receptor binding and internalisation	Prolonged receptor inhibition and delayed-phase protection	[12]
Plasma Half-Life	~9 hours	~40 hours	Extended efficacy with single-dose administration	[27]
Onset of Action	Rapid onset; short duration	Rapid onset; sustained duration	Maintains protection throughout the early and late postoperative phases	[33]
Duration of Efficacy	Effective for early (0–6 h) PONV	Effective for both early and delayed (up to 48 h) PONV	Superior delayed-phase control with palonosetron	[14]
Clinical Efficacy (Middle Ear Surgery)	Granisetron–dexamethasone: ~73% complete response	Palonosetron–dexamethasone: ~97% complete response	Greater efficacy of palonosetron in otologic surgeries	[31]
Binding Affinity (Ki)	Moderate	Highest among 5-HT₃ antagonists	Stronger receptor binding contributes to longer action	[35]
Safety Profile	Mild headache, dizziness; rare QT prolongation	Minimal QT effect; mild transient symptoms	Excellent tolerability for both agents	[40]
Cost-Effectiveness	Lower cost but may require rescue doses	Higher acquisition cost offset by fewer rescues	Cost-effective in prolonged or high-risk surgeries	[19]
Recommended Use	Short-duration or low-risk procedures	High-risk, prolonged, or otologic surgeries	Optimal risk-stratified prophylaxis	[20]

Focus on otologic surgeries

One of the surgical procedures that poses the greatest risk of PONV is the otologic surgeries, namely tympanoplasty and mastoidectomy [17]. This increased vulnerability can be attributed to the fact that during the manipulation of the testes during surgery, the labyrinthine system is stimulated, and emetogenic impulses are relayed to the medullary vomiting center. The smallest amount of stimulation might cause nausea or retching, and the concomitant administration of volatile anesthetics and perioperative opioids increases this risk [22]. In otologic surgery, PONV is not an insignificant ailment; grafts may get dislodged, surgical repairs may be disturbed, and hearing restoration may be severely compromised, especially following tympanoplasty, when surgical repair maintenance is vital to achieving a successful outcome [14]. Therefore, prophylaxis of PONV is crucial not just in order to enhance patient comfort but also to guarantee surgical integrity and long-term outcomes [38].

Although PONV has a clinical significance in otologic surgery, the variety of comparative studies devoted to it is not numerous [45]. There are trials available comparing palonosetron with dexamethasone showing a longer antiemetic coverage than granisetron-dexamethasone [22]. One controlled study showed that palonosetron resulted in a significant reduction in PONV scores in 48 hours and that it reduced the use of rescue antiemetics [36]. Its long half-life (approximately 40 hours) and internalization into the receptors assure persistent receptor blockage in the delayed postoperative period, when PONV relapse is most frequent [26]. On the other hand, the granisetron has a smaller half-life (approximately 9 hours), which allows the drug to treat early emesis but does not provide a sufficiently delayed effect [28]. Palonosetron-dexamethasone gives a distinct clinical benefit in tympanoplasty in cases where vomiting occurs late in the stage, and incorporation of the graft is at risk [41]. Though big multicentric studies are yet to be done to testify on a larger scale, the existing evidence suggests its use as a first-line antiemetic regimen in otologic surgery to ensure stability of the graft and improve recovery after surgery [47].

Safety, tolerability, and adverse effects

A combination of palonosetron-dexamethasone and granisetron-dexamethasone is highly safe and tolerable [10]. Only mild adverse effects, which are mostly headache, dizziness, or temporary constipation, are reported in controlled clinical trials, with no hemodynamic or respiratory complications of any significance [27]. The fact that they do not have any sedative effects makes them particularly appropriate in ambulatory and otologic operations, in which postoperative stability and quick recovery are vital [33].

Additionally, palonosetron is less prone to QT interval prolongation, which is a complication that is sometimes linked with older serotonin antagonists; hence, it is advantageous among patients with other cardiac comorbidities or those taking other drugs that prolong the QT interval [39]. One prophylactic dose of dexamethasone has also been found to be safe, and when used in combination with 5-HT_3_ antagonists, has no additional toxic effect; rather, dual therapy enables lowering of individual dosages, thereby minimizing adverse effects [21]. Other infrequent corticosteroid-induced adverse effects, including transient hyperglycemia or non-immunosuppressive mild immunosuppression, are clinically insignificant at prophylactic levels [35]. Both regimens are very stable in terms of hemodynamics and metabolism, which is crucial in otologic surgeries that need precision of surgery [25]. On the whole, the positive safety profiles of both drug combinations inform the widespread use of both in surgical prophylaxis, with palonosetron-dexamethasone having a slightly better margin of safety of extended protection [30]. Table 3 identifies the relative levels of safety and tolerability of palonosetron-dexamethasone and granisetron-dexamethasone combinations used as PONV prophylaxis.

**Table 3:** Safety and tolerability comparison of palonosetron–dexamethasone and granisetron–dexamethasone combinations 5-HT₃: 5-Hydroxytryptamine type 3, QT: QT interval on electrocardiogram

Safety Parameter	Palonosetron–Dexamethasone	Granisetron–Dexamethasone	Clinical Significance
Common Adverse Effects	Mild headache, dizziness, transient constipation	Similar profile: mild headache, dizziness	Both regimens show excellent tolerability
Hemodynamic Stability	Stable; no significant cardiovascular changes	Stable; no significant cardiovascular changes	Suitable for otologic and ambulatory surgeries
Sedation	None reported	None reported	Ideal for fast recovery and postoperative monitoring
QT Interval Prolongation Risk	Very low; minimal cardiac interaction	Slightly higher risk with older 5-HT₃ agents	Palonosetron preferred for patients with cardiac comorbidities
Corticosteroid-Related Effects	Rare transient hyperglycemia or mild immunosuppression	Similar minimal corticosteroid-related effects	Clinically insignificant at prophylactic doses
Drug Interaction / Toxicity	No increased toxicity when combined with dexamethasone	No increased toxicity when combined with dexamethasone	The combination allows lower dosing and better safety
Overall Safety Rating	Excellent; superior safety margin for prolonged use	Excellent; slightly shorter safety coverage	Both safe; palonosetron–dexamethasone marginally safer

Cost-effectiveness and clinical applicability

There is a rise in economic factors in perioperative pharmacology, particularly where two regimens exhibit similar safety and convenience [19]. The high cost of palonosetron has led to debate around the cost-effectiveness of the drug in general [32]. Nevertheless, when compared to the clinical outcomes, its high effectiveness and extended duration of action frequently command the initial cost [38]. One dose of palonosetron offers 48 hours of PONV prophylaxis, decreasing the necessity of rescue antiemetics and reducing the time of postoperative healing [14]. Consequently, this leads to a reduction in nursing workload and the related healthcare costs, an impact magnified further when indirect savings (including fewer hospital readmissions and better patient satisfaction) are taken into account [27].

The economic argument in favor of the application of palonosetron in otologic surgery is especially strong [40]. A single vomiting episode can lead to graft failure and potentially expensive revision procedures; effective prophylaxis is a cost-effective option [35]. Conversely, granisetron-dexamethasone is still a viable and less expensive option in short or low-risk surgeries, where delayed PONV is not as significant [30]. The choice of the drug should, in this case, also be dependent on the surgical duration, emetogenic risk, and institutional cost structure and not on the acquisition price itself [16]. Both of these regimens are well tolerated, predictable pharmacologically, and can be used with standard anesthesia procedures, which support their usefulness in a wide variety of surgical procedures [25].

Evidence synthesis and gaps in the literature

The overall evidence suggests that both regimens can be used to prevent PONV; nevertheless, palonosetron-dexamethasone is always found to have better efficacy during the delayed postoperative period, especially in high-risk surgeries like middle ear surgery [27]. Its long receptor affinity and long half-life result in reduced rescue antiemetic, increased patient comfort, and improved surgical safety [33]. Conversely, granisetron-dexamethasone continues to be a worthy option for short-acting, still cost-efficient, and accessible to lower-risk or shorter surgeries [42]. Although these results are positive, there are still a number of research gaps [21]. The available evidence is based on most of the small and single-center trials, which restrict the ability to generalize the results to a large and diverse patient group and surgical scenarios [39]. Large, multicentric, randomized controlled trials are required to confirm the existing findings [35]. Besides, there is limited information regarding pediatric and geriatric populations, yet these populations frequently display different pharmacokinetic characteristics and extreme sensitivity to emetogenic stimuli [43].

Economic assessments should also be investigated further since cost-utility analysis, including the length of stay in the hospital, patient satisfaction, and avoidance of surgical complications, is insufficiently covered [28]. Similarly, studies investigating dexamethasone in extended multimodal regimens, such as triple therapy with neurokinin-1 (NK1) receptor antagonists, would further reduce PONV in ultra-high-risk groups [30]. The future directions should focus on personalizing antiemetic prophylaxis, combining genetic polymorphisms associated with serotonin receptor sensitivity and metabolism, and predicting risks in the context of individualized treatment decisions [40]. These methods offer better efficacy and cost-effectiveness in the perioperative environment [24].

To conclude, existing evidence confirms that both combinations are safe, flexible, and effective in operative situations, although palonosetron-dexamethasone offers greater, more consistent protection, which is one of the benefits in otologic surgery where vomiting may put the integrity of the surgery at risk [45]. To enhance guidelines that are evidence-based and high-quality, rigorously designed studies with standardized outcomes and sound economic evaluations are needed to promote fair and cost-effective application through healthcare systems [34].

Limitations and future recommendations

The limitations of the existing literature include a paucity of high-quality evidence on antiemetic prophylaxis in otologic surgery, despite encouraging preliminary results. Comparative evaluations of palonosetron with dexamethasone versus granisetron with dexamethasone are limited to a small number of single-center studies with modest sample sizes and short follow-up durations of 24-48 hours. Such study designs preclude reliable assessment of delayed PONV, optimal dosing strategies, and long-term safety. Furthermore, the lack of large multicenter randomized controlled trials reduces external validity, and the near absence of cost-effectiveness analyses and patient-reported outcome measures limits clinically and economically meaningful interpretation, particularly in high-risk otologic procedures where postoperative vomiting can jeopardize surgical outcomes.

In combination with this, cost and cost-effectiveness analysis may play a significant role in determining the true value of the use of long-acting agents such as palonosetron [10]. In addition, additional drug combinations, such as NK1 receptor antagonist drugs or triple drug regimens as an extension of prophylaxis, could be investigated to enhance prophylaxis. Finally, integration of individualized methods, buttressed by clinical risk scoring systems and pharmacogenomic profiling, will have the potential to assist in the customization of the antiemetic choice, thereby achieving the maximum possible efficacy, and allocation of resources will be made effective, including the provision of safer and more patient-focused perioperative care.

## Conclusions

This narrative review represents a unique intersection and integration of the comparative effectiveness of palonosetron-dexamethasone and granisetron-dexamethasone combinations into the narrower scope of otologic surgery, an area in which procedure-specific comparative evidence remains relatively limited despite the widespread mainstream use of 5-HT₃ antagonists with dexamethasone in modern anesthesia practice. It combines pharmacologic evidence with procedure-specific clinical experience, illustrating the role of the vestibular stimulation that accompanies middle ear surgery, increasing the necessity of long-acting antiemetic coverage. The synthesis illustrates that both regimens are effective and tolerable, but that palonosetron has a higher receptor affinity, prolonged half-life, and longer action, which makes it especially beneficial in the prevention of postoperative emesis delays and graft integrity. As opposed to the previous generalized reviews, this discussion identifies the clinical, economic, and procedural peculiarities of otologic settings and provides an evidence-based argument in favor of risk-stratified prophylaxis. It highlights the necessity of using serotonin receptor blockers with corticosteroids to achieve synergistic and long-term protection and suggests that multicentric, cost-effective, and personalized studies are necessary to streamline future recommendations. Rather than redefining established antiemetic principles, this review strengthens their otologic applicability by consolidating available head-to-head evidence and highlighting priorities for future procedure-specific trials.

## References

[1] Liu HM, Chen JH, Chen C, Liou CM (2021). Prophylactic antiemetic effects of dexamethasone versus 5-HT3 receptor antagonists in ear surgery: a systematic review and meta-analysis. Int J Clin Pharm.

[2] Tricco AC, Blondal E, Veroniki AA (2016). Comparative safety and effectiveness of serotonin receptor antagonists in patients undergoing chemotherapy: a systematic review and network meta-analysis. BMC Med.

[3] Kovac AL (2016). Comparative pharmacology and guide to the use of the serotonin 5-HT3 receptor antagonists for postoperative nausea and vomiting. Drugs.

[4] Meyer TA, Hutson LR Jr, Morris PM, McAllister RK (2023). A postoperative nausea and vomiting update: current information on new drugs, old drugs, rescue/treatment, combination therapies and nontraditional modalities. Adv Anesth.

[5] Bauiomy H, Kohaf NA, Saad M (2025). Study the effect of intraperitoneal dexamethasone, dexmedetomidine, and their combination on PONV after laparoscopic cholecystectomy: a randomized triple-blind trial. Anesthesiol Res Pract.

[6] Bhakta A, Goel R (2017). Causes and treatment of nausea and vomiting. Prescriber.

[7] Apfel CC, Läärä E, Koivuranta M (1999). A simplified risk score for predicting postoperative nausea and vomiting: conclusions from cross-validations between two centers. Anesthesiology.

[8] Divatia JV, Vaidya JS, Badwe RA, Hawaldar RW (1996). Omission of nitrous oxide during anesthesia reduces the incidence of postoperative nausea and vomiting. A meta-analysis. Anesthesiology.

[9] Peyton PJ, Wu CYX (2014). Nitrous oxide-related postoperative nausea and vomiting depends on duration of exposure. Sur Anes.

[10] Pace NL (2014). Questioning a relationship between nitrous oxide duration of exposure and postoperative nausea and vomiting. Anesthesiology.

[11] Honarmand A, Safavi M, Chegeni M (2016). Prophylactic antiemetic effects of Midazolam, Ondansetron, and their combination after middle ear surgery. J Res Pharm Pract.

[12] Livingstone DM, Smith KA, Lange B (2017). Scuba diving and otology: a systematic review with recommendations on diagnosis, treatment and post-operative care. Diving Hyperb Med.

[13] Tolvi M, Lehtonen L, Tuominen-Salo H (2021). Overstay and readmission in ear, nose, and throat day surgery—factors affecting postanesthesia course. Ear Nose Throat J.

[14] Sahni N, Panda N, Kumar A (2022). Comparison of palonosetron with combination of palonosetron and dexamethasone in the prevention of postoperative nausea and vomiting in patients undergoing middle ear surgery: a prospective randomized trial. Indian J Otolaryngol Head Neck Surg.

[15] Morrison DR, Moore LS, Walsh EM (2020). Perioperative pain management following otologic surgery. Otolaryngol Clin North Am.

[16] Bao PH, Friedland DR, Adams JA (2025). Assessment of anesthetic modalities in otologic surgery. Otol Neurotol Open.

[17] Chorath K, Hobday S, Suresh NV (2022). Enhanced recovery after surgery protocols for outpatient operations in otolaryngology: Review of literature. World J Otorhinolaryngol Head Neck Surg.

[18] Tan JQ, Chen YB, Wang WH (2021). Application of enhanced recovery after surgery in perioperative period of tympanoplasty and mastoidectomy. Ear Nose Throat J.

[19] Schmidt AP (2020). Prevention of postoperative nausea and vomiting: new insights for patient care. Braz J Anesthesiol.

[20] Elvir-Lazo OL, White PF, Yumul R, Cruz Eng H (2020). Management strategies for the treatment and prevention of postoperative/postdischarge nausea and vomiting: an updated review. F1000Res.

[21] Kovac AL (2025). Pathophysiology and risk factors for postoperative nausea and vomiting in adults and children. BJA Educ.

[22] Zhang Z, Wang X (2025). The neural mechanism and pathways underlying postoperative nausea and vomiting: a comprehensive review. Eur J Med Res.

[23] Bacho MT, Wolda GD, Demssie WR (2025). Incidence and risk factors of postoperative nausea and vomiting in Africa among patient under gone surgery: a systematic review and meta-analysis. Ann Med Surg (Lond).

[24] Awad K, Ahmed H, Abushouk AI (2016). Dexamethasone combined with other antiemetics versus single antiemetics for prevention of postoperative nausea and vomiting after laparoscopic cholecystectomy: An updated systematic review and meta-analysis. Int J Surg.

[25] Mohd DFN, Seevaunnamtum SP, Mohamad NNA (2021). Granisetron vs. granisetron and dexamethasone on the reduction of postoperative nausea and vomiting (PONV) after caesarean section with intrathecal morphine: a randomised controlled trial. Egypt J Anaesth.

[26] Sadhoo N, Prakash J, Kumar V (2023). A comparison of prophylactic antiemetic therapy with palonosetron and dexamethasone as single-agents or in combination in adult patients undergoing laparoscopic surgery: A randomized trial. Int J Crit Illn Inj Sci.

[27] Jain P, Ahluwalia P, Ahluwalia A, Jain R (2019). Study comparing high dose palanosetron 0.075 mg with low dose palanosetron 0.05 mg plus 4mg dexamethasone as adjuvant for prevention of post-operative nausea and vomiting in laproscopic hysterectomies-a double blinded study. Ann Advan Nurs.

[28] Sharma AN, Shankaranarayana P (2015). Postoperative nausea and vomiting: palanosetron with dexamethasone vs. ondansetron with dexamethasone in laparoscopic hysterectomies. Oman Med J.

[29] Chatterjee A, Sahu S, Paul M (2017). Comparison of efficacy of palonosetron-dexamethasone combination with palonosetron or dexamethasone alone for prophylaxis against post-operative nausea and vomiting in patients undergoing laparoscopic cholecystectomy. Indian J Anaesth.

[30] Rajnikant K, Bhukal I, Kaloria N (2019). Comparison of palonosetron and dexamethasone with ondansetron and dexamethasone to prevent postoperative nausea and vomiting in patients undergoing laparoscopic cholecystectomy. Anesth Essays Res.

[31] Zhu M, Zhou C, Huang B (2017). Granisetron plus dexamethasone for prevention of postoperative nausea and vomiting in patients undergoing laparoscopic surgery: a meta-analysis. J Int Med Res.

[32] Prerna Prerna, Chadha J, Khullar L (2024). A comprehensive review on the pharmacological prospects of Terpinen-4-ol: From nature to medicine and beyond. Fitoterapia.

[33] Nanjundaswamy NH, Sridhara RB (2018). A comparative study of ondansetron and granisetron in combination with dexamethasone in prophylaxis for postoperative nausea and vomiting (PONV) in laparoscopic cholecystectomies. Int J Res Med Sci.

[34] Gugale AA, Bhalerao PM (2016). Palonosetron and granisetron in postoperative nausea vomiting: A randomized double-blind prospective study. Anesth Essays Res.

[35] A Mahrous M, A El-Azab G, A Tawfik H (2021). Evaluation of clinical outcomes and efficacy of palonosetron and granisetron in combination with dexamethasone in Egyptian patients receiving highly emetogenic chemotherapy. Cancer Chemother Pharmacol.

[36] Narayanappa AB, Gurulingaswamy S, Prabhakaraiah UN (2017). Intravenous palonosetron compared with a combination of ramosetron and dexamethasone in preventing post operative nausea and vomiting in patients undergoing gynaecological surgeries under spinal anaesthesia, a randomised study. Indian J Anaesth.

[37] Sharma SK, Singh S, Paul D (2020). Effect of dexamethasone with granisetron or ondansetron for prevention of post-operative nausea vomiting in patients undergoing laparoscopic gynaecological surgery. Int J Res Med Sci.

[38] Vishwasrao SS, Vishwasrao SM, Kumar AN (2025). Comparative study of intravenous granisetron against ondansetron in preventing postoperative nausea vomiting in patients undergoing general anaesthesia: a double-blinded randomised controlled study. J Clin Diagn Res.

[39] Makwana J, Vadodariya D, Vadodariya B (2017). Palonosetron and granisetron for the prevention of postoperative nausea and vomiting after laparoscopic cholecystectomy: a prospective comparative randomised active controlled trial. J Med Sci Clin Res.

[40] Gouveia de Araujo Ferreira N, Cavalcanti IL, Assad AR (2020). A prospective, randomized, double-blind trial to compare body weight-adjusted and fixed doses of palonosetron for preventing postoperative nausea and vomiting in obese female patients. PLoS One.

[41] Chadha J, Ahuja P, Mudgil U (2024). Citral and triclosan synergistically silence quorum sensing and potentiate antivirulence response in Pseudomonas aeruginosa. Arch Microbiol.

[42] Kovac AL (2018). Updates in the management of postoperative nausea and vomiting. Adv Anesth.

[43] Hong JM, Han YH, Lee D (2021). Comparison of efficacy between palonosetron-midazolam combination and palonosetron alone for prevention of postoperative nausea and vomiting in patients undergoing breast surgery and patient controlled analgesia: A prospective, randomized, double-blind study: A CONSORT-compliant study. Medicine (Baltimore).

[44] Swaro S, Karan D, Banerjee A (2018). Comparison of palonosetron, dexamethasone, and palonosetron plus dexamethasone as prophylactic antiemetic and antipruritic drug in patients receiving intrathecal morphine for lower segment cesarean section. Anesth Essays Res.

[45] Chitta P, Mothe G, Alugolu M, Leela KS (2022). Efficacy of ondansetron alone, dexamethasone alone and combination of ondansetron and dexamethasone for PONV for patients undergoing under general anaesthesia. Int J Health Sci.

[46] Grigio TR, Sousa AM, Magalhães GG (2020). Aprepitant plus palonosetron for the prevention of postoperative nausea and vomiting after breast cancer surgery: a double blind, randomized trial. Clinics (Sao Paulo).

[47] Som A, Bhattacharjee S, Maitra S (2016). Combination of 5-HT3 antagonist and dexamethasone is superior to 5-HT3 antagonist alone for PONV prophylaxis after laparoscopic surgeries: a meta-analysis. Anesth Analg.

[48] Dey S, Chanu SM, Dev P (2021). Antiemetic efficacy of palanosetron compared with the combination of ondansetron and dexamethasone for prevention of postoperative nausea and vomiting in patients undergoing laparoscopic gynaecological surgery. Rom J Anaesth Intensive Care.

